# Minimally Invasive Coronary Artery Revascularization Surgery Versus Conventional Techniques in Patients With Complex Coronary Artery Disease: A Systematic Review of Cardiac Function

**DOI:** 10.7759/cureus.102275

**Published:** 2026-01-25

**Authors:** Paulina Elizabeth Cisneros Clavijo, Andrés Sebastian Moreno Barragan, Mariana López Hernández, John Manuel Dorado Ramírez, Laura Camila Sanchez Prada, Nicolas David Bonilla Rojas, Sergio Daniel Zabaleta Orozco

**Affiliations:** 1 Endovascular Surgery, Enrique Garcés Hospital, Quito, ECU; 2 Especialista en Hemodinamia y Cardioangiologia general e intervencionista., Pontificia Universidad Catolica del Ecuador, Quito, ECU; 3 Medical Department, Universidad Autónoma de los Andres, Riobamba, ECU; 4 Medical Department, Universidad Ciencias de la Salud, Medellín, COL; 5 Internal Medicine, Instituto Mexicano del Seguro Social, Mexico City, MEX; 6 Medical Department, Universidad Autonoma de Bucaramanga, Bucaramanga, COL; 7 Medical Department, Universidad Industrial de Santander, Bucaramanga, COL; 8 Medical Department, Universidad Surcolombiana, Neiva, COL

**Keywords:** a systematic review, cardiac function, coronary artery disease, coronary artery revascularization, minimally invasive

## Abstract

Coronary artery disease (CAD) requiring multivessel revascularization, commonly defined by involvement of the left anterior descending artery plus ≥1 major epicardial vessel, left-main disease, or elevated anatomic complexity such as higher SYNTAX scores, is traditionally managed with conventional sternotomy coronary artery bypass grafting (CABG), but its invasiveness has driven interest in minimally invasive alternatives. This systematic review compares cardiac outcomes of minimally invasive coronary revascularization versus conventional CABG in patients with multivessel or complex CAD. The review followed Preferred Reporting Items for Systematic Reviews and Meta-Analyses (PRISMA) guidelines and searched PubMed/MEDLINE, Google Scholar, the Cochrane Library, and ScienceDirect from inception to November 2025. Minimally invasive techniques included minimally invasive direct CABG (MIDCAB), minimally invasive multivessel CABG (MICS-CABG), endoscopic or robotic-assisted CABG (TECAB/Endo-CAB), and hybrid coronary revascularization (HCR) combining minimally invasive LIMA-left anterior descending (LAD) grafting with percutaneous coronary intervention (PCI). Comparative studies versus conventional sternotomy CABG were eligible. Two reviewers independently performed study selection, data extraction, and quality assessment using ROB 2.0 for randomized controlled trials (RCTs) and ROBINS-I for observational studies. Nineteen studies were included, comprising a small number of RCTs and predominantly propensity-matched or retrospective comparative cohorts, which informed confidence in findings. Due to substantial heterogeneity in surgical approaches, patient selection, outcome definitions, and follow-up duration, results were synthesized narratively.

Across the included evidence, minimally invasive coronary revascularization demonstrated cardiac outcomes comparable to conventional CABG in complex CAD patients. Early (in-hospital or 30-day) and mid-term (1-5 year) mortality remained low and similar between groups, typically ranging from 0.5-3%. Rates of myocardial infarction and stroke were likewise comparable, generally within 1-4% and 0.5-2%, respectively. Composite major adverse cardiac/cerebrovascular events (MACCE/MACE) outcomes were equivalent across approaches, most commonly 5-12%. Atrial fibrillation was less frequent in several endoscopic or highly minimally invasive cohorts, though reductions were not uniform across all techniques. Repeat revascularization ranged from 2-7%, with slightly higher rates observed in some HCR cohorts, likely reflecting PCI durability rather than failure of the surgical LIMA-LAD graft. Perioperative outcomes consistently favored minimally invasive approaches, including reduced blood loss, shorter ventilation times, and decreases of 0.5-2 days in ICU stay and 2-4 days in total hospital stay. Standardized cardiac functional measures, such as LVEF or heart failure class, were inconsistently reported and therefore not pooled.

Overall, minimally invasive coronary revascularization provides major clinical outcomes comparable to conventional CABG while offering meaningful reductions in perioperative morbidity and faster recovery, supporting its selective use in appropriately chosen complex CAD patients, particularly in experienced, high-volume centers.

## Introduction and background

Coronary artery disease (CAD) is the leading cause of death and disability in developed countries and is associated with approximately 17.8 million deaths annually worldwide [1]. Revascularization strategies for patients with multivessel or complex CAD primarily include percutaneous coronary intervention (PCI) and coronary artery bypass grafting (CABG). Multivessel CAD is defined as the presence of significant stenoses (≥70%) in two or more major epicardial coronary arteries, while complex CAD refers to anatomically high-risk disease, including left-main coronary artery involvement and/or moderate-to-high anatomical complexity as reflected by elevated SYNTAX scores (typically ≥23), in line with contemporary guideline-based classifications.

PCI represents a non-surgical revascularization strategy with advantages such as shorter procedural time and less complicated post-revascularization recovery, including reduced infection risk and transfusion requirements [2]. However, in patients with multivessel or complex CAD, PCI is associated with higher rates of repeat revascularization and, in selected high-risk anatomical subgroups, less favorable long-term outcomes when compared with CABG, despite comparable short-term mortality [3]. In contrast, CABG is a surgical revascularization procedure traditionally performed via a median sternotomy, with thoracotomy-based approaches reserved for minimally invasive techniques rather than conventional surgery, and may be conducted with or without cardiopulmonary bypass [4].

CABG remains the cornerstone treatment for patients with complex multivessel disease, particularly those with left-main or triple-vessel involvement, offering superior long-term survival and lower rates of repeat revascularization compared with medical therapy or PCI alone in appropriately selected patients [5]. The standard CABG approach-median sternotomy with cardiopulmonary bypass (on-pump CABG)-provides durable long-term graft patency, especially when the left internal thoracic artery (LITA) is anastomosed to the left anterior descending (LAD) artery [6].

Nevertheless, the invasiveness of conventional sternotomy-based CABG is associated with important limitations, including prolonged recovery, increased surgical trauma, bleeding and transfusion requirements, sternal wound complications, and extended intensive care unit (ICU) and hospital stays [7]. To mitigate these disadvantages, minimally invasive coronary revascularization strategies have been developed. In this review, minimally invasive coronary surgery (MICS) is defined as CABG performed without full median sternotomy, including minimally invasive direct CABG (MIDCAB) via limited thoracotomy, endoscopic or robotic-assisted approaches, and related port-access techniques. Importantly, off-pump CABG performed through full sternotomy is classified as a conventional surgical approach and is analytically distinct from minimally invasive off-pump techniques. These approaches frequently avoid cardiopulmonary bypass and aim to reduce procedural morbidity [8].

Originally developed for isolated LAD revascularization, MICS techniques have progressively evolved to permit multivessel bypass grafting, expanding their applicability to patients with more complex coronary anatomy beyond single-vessel disease [9-10]. This evolution has enabled minimally invasive strategies to be considered for selected patients traditionally referred for conventional CABG.

In parallel, hybrid coronary revascularization (HCR) emerged in the mid-1990s as a combined approach involving minimally invasive LITA-LAD bypass grafting with PCI using drug-eluting stents for non-LAD lesions [11]. This strategy seeks to combine the long-term durability of surgical LAD revascularization with the reduced invasiveness and faster recovery associated with PCI, potentially minimizing surgical trauma while maintaining acceptable completeness of revascularization [12].

Several systematic reviews have evaluated individual minimally invasive coronary techniques; however, each provides a limited or fragmented perspective. Florisson et al. compared MIDCAB with off-pump sternotomy-based CABG and demonstrated shorter ICU and hospital stays with MIDCAB, albeit with higher rates of incomplete revascularization, while mortality remained similar [13]. Dixon et al. examined HCR versus conventional CABG in multivessel disease, reporting comparable mid-term survival and MACE, reduced transfusion requirements, and shorter ICU stays, but with a trend toward higher early mortality [14]. Alsharif et al. conducted a broad review comparing minimally invasive and open CABG but highlighted substantial heterogeneity in patient selection, coronary anatomical complexity, surgical techniques, operator experience, and outcome definitions, limiting the applicability of their findings to patients with complex or multivessel CAD [15].

Notably, prior reviews have seldom integrated the full spectrum of minimally invasive strategies, including multivessel MICS, robotic-assisted CABG, and hybrid revascularization, nor have they consistently focused on clinically meaningful cardiac outcomes such as mortality, myocardial infarction, stroke, atrial fibrillation, repeat revascularization, and composite major adverse cardiac/cerebrovascular events (MACCE/MACE), which directly reflect procedural safety, durability, and long-term cardiovascular risk. Moreover, outcomes of MICS and HCR are known to be influenced by surgeon expertise and institutional procedural volume, factors that may contribute to inter-study variability and are infrequently addressed in prior syntheses.

Accordingly, the present systematic review aims to inform comparative effectiveness and surgical decision-making by systematically synthesizing cardiac and perioperative outcomes associated with minimally invasive coronary revascularization techniques versus conventional sternotomy-based CABG, specifically in patients with multivessel or complex CAD.

## Review

Methods

Study Design

This study was conducted as a systematic review evaluating cardiac functional outcomes, defined primarily as clinically relevant cardiac event-based outcomes reflecting functional cardiovascular status and durability of revascularization, and related perioperative endpoints in patients with complex CAD undergoing minimally invasive coronary revascularization surgery compared with conventional sternotomy-based CABG. Cardiac functional outcomes were distinguished from purely procedural metrics and included mortality, myocardial infarction, stroke, atrial fibrillation, repeat revascularization, graft patency, and composite MACCE/MACE; surrogate measures such as left ventricular ejection fraction or functional class were not required for study inclusion and were recorded only when incidentally reported. The review followed the Preferred Reporting Items for Systematic Reviews and Meta-Analyses (PRISMA) guidelines [15].

Search Strategy

A comprehensive literature search was conducted across PubMed/MEDLINE, Google Scholar, the Cochrane Library, and ScienceDirect from inception to November 2025. The search strategy combined controlled vocabulary terms and free-text keywords related to minimally invasive coronary surgery (including MIDCAB, MICS-CABG, TECAB, robot-assisted CABG, and hybrid coronary revascularization), conventional CABG, multivessel or complex coronary artery disease, SYNTAX score, and cardiac outcomes such as mortality, myocardial infarction, stroke, atrial fibrillation, repeat revascularization, graft patency, and MACCE/MACE. For Google Scholar, the first 200 search results-a commonly accepted reproducible screening threshold-were reviewed for eligibility. Additionally, reference lists of all included studies and relevant systematic reviews were manually screened to ensure comprehensive coverage.

Only studies published in English were considered. No restrictions were placed on publication date. Conference abstracts, unpublished data, trial registries, and other grey literature sources were not systematically searched due to limited outcome reporting and concerns regarding data completeness.

Eligibility Criteria

Studies were included if they enrolled adult patients with multivessel or complex CAD, such as involvement of the LAD artery plus at least one additional major epicardial vessel, patients with elevated SYNTAX scores, or individuals eligible for multivessel revascularization. Eligible interventions encompassed any minimally invasive coronary revascularization approach, including minimally invasive direct CABG (MIDCAB), minimally invasive multivessel CABG (MICS-CABG), totally endoscopic or robot-assisted CABG (TECAB/RACAB), and HCR, defined as LIMA-LAD grafting via a minimally invasive approach combined with PCI. The comparator was conventional full sternotomy CABG, performed either on-pump or off-pump.

Studies were required to report at least one predefined cardiac outcome (mortality, myocardial infarction, stroke, atrial fibrillation, repeat revascularization, graft patency, or composite MACCE/MACE). Quality-of-life or functional recovery outcomes (e.g., angina status, activities of daily living, or return to work) were considered secondary outcomes and were included when reported, without being mandatory for study inclusion. Eligible study designs included randomized controlled trials (RCTs), prospective cohorts, retrospective matched cohorts, and propensity-score-adjusted studies. Exclusion criteria comprised case reports, non-comparative cohorts, pediatric populations, studies focusing exclusively on isolated single-vessel LAD disease, and purely technical or feasibility reports without clinical outcomes.

Study Selection

All retrieved records were imported into a reference management system, and duplicates were removed. Two reviewers independently screened titles and abstracts to identify potentially eligible studies. Full texts of shortlisted articles were then assessed in detail against the predefined inclusion and exclusion criteria. Disagreements were resolved through discussion; when consensus could not be achieved, a third reviewer adjudicated the decision.

In cases where multiple publications appeared to derive from overlapping patient populations, study characteristics, enrollment periods, and institutional sources were compared, and the most comprehensive or recent dataset was included to avoid double-counting.

Data Extraction

Data extraction was performed independently by two reviewers using a standardized data extraction form. Extracted information included study design, publication year, sample size, CAD complexity measures, and details of minimally invasive and conventional interventions. Outcomes of interest included cardiac endpoints (mortality, myocardial infarction, stroke, atrial fibrillation, repeat revascularization, graft patency, and composite MACCE/MACE), as well as perioperative and recovery parameters (operative duration, blood loss, transfusion requirements, ventilation time, ICU stay, and total hospital length of stay). Quality-of-life and functional recovery outcomes (e.g., angina-free survival, activities of daily living scores) were recorded when explicitly reported, without imposing restrictions on specific measurement instruments. When available, statistical indicators such as effect sizes, p-values, and 95% confidence intervals were extracted. Missing or incompletely reported outcome data were not imputed and were reported narratively as unavailable. Any discrepancies were resolved by consensus. Outcome definitions were accepted as reported in the original studies and were not redefined or harmonized across datasets.

Risk of Bias Assessment

Risk of bias was independently assessed by two reviewers using validated tools appropriate to the study design. Randomized controlled trials (RCTs) were evaluated using the Cochrane Risk of Bias 2.0 (ROB 2.0) tool, while observational studies were assessed using the Risk Of Bias In Non-randomized Studies of Interventions (ROBINS-I) instrument [16]. Domains assessed included confounding, selection bias, classification of interventions, deviations from intended interventions, missing data, outcome measurement, and selective reporting. Each study was assigned an overall judgment of low, moderate, serious, or critical risk of bias. Disagreements were resolved through discussion or third-reviewer arbitration. Formal certainty-of-evidence grading (e.g., GRADE) was not planned due to substantial clinical and methodological heterogeneity and the narrative nature of the synthesis.

Data Synthesis

A narrative synthesis approach was adopted owing to significant methodological and clinical heterogeneity across studies. Variability was observed in minimally invasive techniques (MIDCAB, MICS-CABG, robotic-assisted CABG, endoscopic CABG, and HCR), comparator interventions (on-pump and off-pump sternotomy CABG), patient populations, outcome definitions, and follow-up durations. Heterogeneity was assessed qualitatively by examining differences in study design, patient selection, CAD complexity, surgical approach, outcome definitions, and timing of outcome assessment. Given these differences, quantitative meta-analysis was not performed.

Findings from all eligible studies were synthesized descriptively. Cardiac outcomes-mortality, myocardial infarction, stroke, atrial fibrillation, repeat revascularization, and composite MACCE/MACE-were summarized narratively and tabulated with reported statistics. Perioperative, recovery, and quality-of-life outcomes were similarly synthesized. Reported effect measures were presented exactly as described in the original publications, without recalculation or harmonization of outcome definitions.

Results

**Figure 1:**
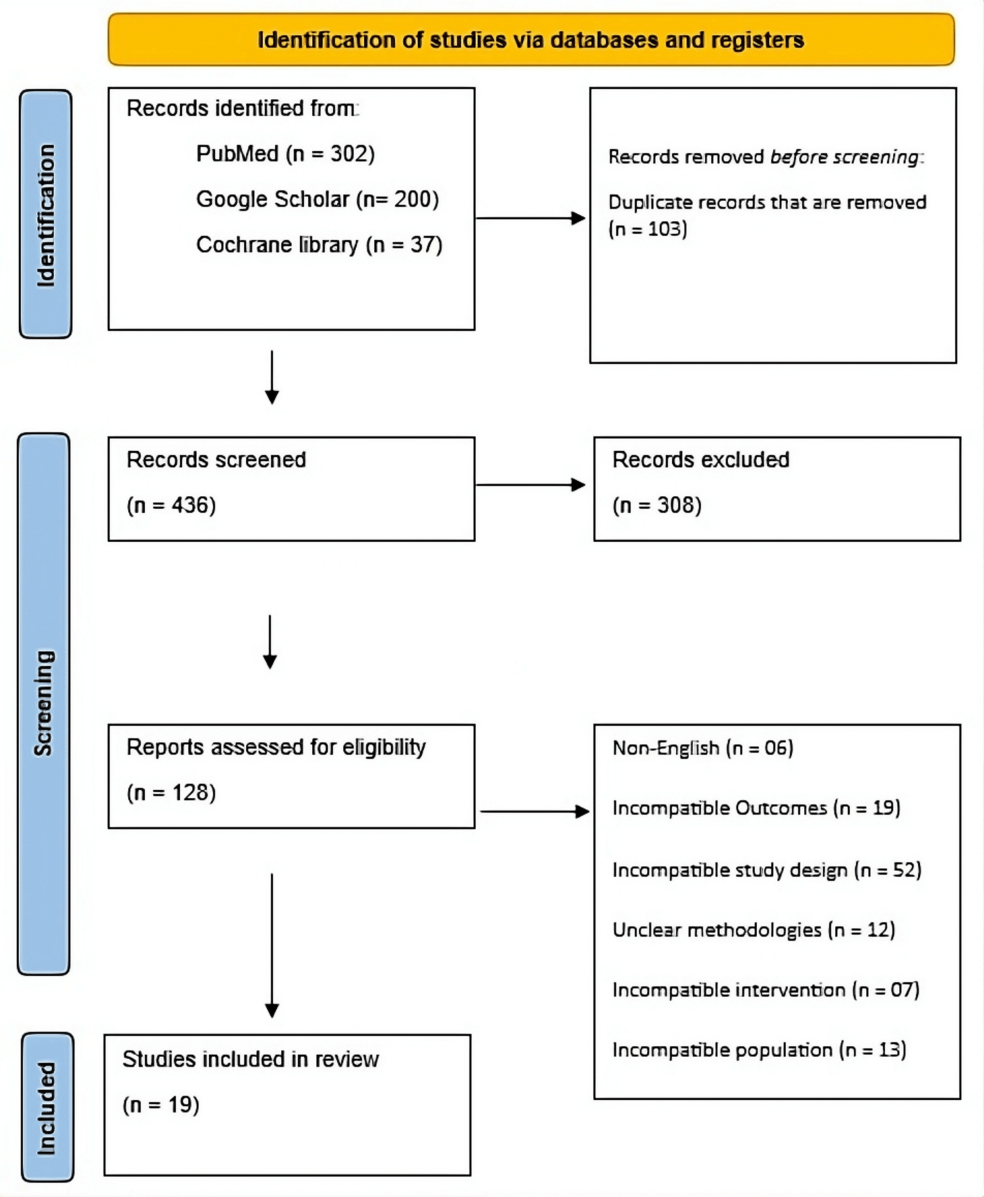
PRISMA flow diagram. The flow diagram illustrates the study selection process: number of records identified, screened, excluded, and included in the review. Reasons for exclusions of full-text articles are indicated, showing transparency in study selection.The flow diagram illustrates the study selection process: number of records identified, screened, excluded, and included in the review. Reasons for exclusions of full-text articles are indicated, showing transparency in study selection.

The PRISMA flow diagram outlines the study selection process for the review. A total of 539 records were initially identified through database searches, including PubMed (n = 302), Google Scholar (n = 200), and the Cochrane Library (n = 37). After removing 103 duplicate records before screening, 436 records proceeded to the screening stage. Of these, 308 were excluded based on title and abstract review, leaving 128 full-text articles assessed for eligibility. Following detailed evaluation, several studies were excluded due to reasons such as being non-English (n = 6), incompatible outcomes (n = 19), inappropriate study design (n = 52), unclear methodologies (n = 12), incompatible interventions (n = 7), and mismatched populations (n = 13). Ultimately, 19 studies met all inclusion criteria and were incorporated into the final review (Figure 1).

Study Characteristics

This review included 19 studies published between 2011 and 2025, comprising RCTs, prospective comparative studies, and predominantly retrospective matched cohort analyses. Sample sizes varied widely, from small pilot RCTs such as the MERGING trial (n=60) and the HREVS trial (n=155), to large-scale observational cohorts exceeding 1,900 patients. Most studies focused on patients with multivessel or complex coronary artery disease, frequently classified using SYNTAX scores or anatomic criteria (LAD involvement plus ≥1 additional major vessel). The minimally invasive interventions evaluated included MIDCAB, MICS-CABG, robotic-assisted MIDCAB, endoscopic CAB, and various forms of HCR combining LIMA-LAD grafting with PCI to non-LAD vessels. Comparator groups primarily consisted of conventional off-pump or on-pump CABG via median sternotomy, though several studies also compared HCR against multi-vessel PCI. Propensity-score matching was employed in most observational studies to balance baseline characteristics. Across studies, populations were heterogeneous, including diabetic cohorts, elderly subgroups, SYNTAX-stratified patients, and candidates for total arterial multivessel revascularization (Table 1).

**Table 1:** Characteristics and results of the studies included. HCR: hybrid coronary revascularization (LIMA-LAD + PCI to non-LAD vessels); MIDCAB: minimally invasive direct CABG; MICS-CABG: minimally invasive CABG; OPCAB: off-pump CABG; CABG: conventional coronary artery bypass; PCI: percutaneous coronary intervention; LITA: left internal thoracic artery; LAD: left anterior descending artery; MVCAD: multivessel coronary artery disease; DES: drug-eluting stent; MACCE/MACE: major adverse cardiac and cerebrovascular events; OR: operating room; LOS: length of stay; ICU: intensive care unit; PSM: propensity score matching; SYNTAX: anatomical complexity score; AF: atrial fibrillation Metrics: Study design, population, CAD complexity, intervention/control, cardiac outcomes (mortality, MI, stroke, AF, revascularization, MACCE/MACE), and high-level perioperative or functional findings.

Author/Year	Study Design	Population (Type & N)	Multivessel / Complex CAD?	Intervention	Control	Cardiac Outcomes	Other Key Findings (High-Level Summary)
Giambruno et al. 2017 [17]	Retrospective cohort, propensity weighted	On-pump CABG n=682; HCR n=147	Multivessel CAD (LAD + one vessel)	Robotic MIDCAB (LITA-LAD) + PCI	On-pump CABG	In-hospital: similar re-exploration, MI, stroke, AF; slightly higher early death in HCR; Long-term: survival similar, freedom from revascularization similar, lower freedom from angina in HCR	Shorter LOS; lower prolonged ventilation; no differences in dialysis or transfusion.
Zhang et al. 2016 [18]	Retrospective observational	MIDCAB n=300; OPCAB n=355	Mixed: single LAD, hybrid MVD, incomplete MVD	MIDCAB (mixed: hybrid 163, single LAD 93, MVD incomplete 44)	Off-pump CABG via sternotomy	Postop MI, re-exploration, 30-day mortality all similar; early graft occlusion rare; overall cardiac outcomes comparable	Shorter OR time, ventilation duration, ICU stay, and fewer transfusion units.
Lapierre et al. 2011 [19]	Case-matched cohort	300 patients (150 MICS-CABG via small thoracotomy vs 150 OPCAB),	Yes – multivessel CABG candidates	Minimally invasive CABG via small thoracotomy (MICS-CABG)	Off-pump CABG via sternotomy (OPCAB)	Mortality similar; AF and reoperation rates comparable; no difference in anastomotic revision	Shorter hospital stay, lower wound infection, higher pleural effusion, faster functional recovery, and a small conversion rate.
Halkos et al. 2011 [20]	Retrospective matched cohort (4:1 matching)	735 patients (147 HCR vs 588 OPCAB),	Yes – multivessel CAD	Hybrid coronary revascularization (minimally invasive LIMA-LAD + PCI to non-LAD)	Off-pump CABG via sternotomy (OPCAB)	In-hospital death, stroke, MI, MACCE similar; long-term repeat revascularization higher in HCR; otherwise, outcomes comparable	Lower transfusion rate; similar LOS, AF, and ventilation hours; similar 5-year survival.
Guangxin et al., 2024 [21]	Single-center retrospective, PSM 1:1	104 patients (52 MICS-CABG, 52 CCABG) with CAD + diabetes, 2017–2021	Mixed CAD with diabetes, not strictly multivessel	MICS-CABG (transthoracic minimally invasive CABG)	Conventional CABG (sternotomy)	MACCE, mortality, MI, stroke, repeat revascularization, graft patency, all similar between MICS-CABG and conventional CABG	Longer surgery time; slightly less blood loss; fewer wound debridements; similar graft quality.
Liang et al., 2022 [22]	Retrospective, PSM 1:1, SYNTAX-based matching	344 matched patients (172 MICS-CABG, 172 OPCABG), 2017–2021	Multivessel CAD, SYNTAX-based	MICS-CABG (minimally invasive approach)	Off-pump CABG (sternotomy OPCABG)	30-day mortality, MI, stroke, reoperation, AF, transfusion all similar; early cardiac outcomes comparable	Shorter LOS, better postoperative ADL scores, and similar graft patency.
Gong et al., 2025 [23]	Retrospective, PSM 1:1	488 patients (244 MICS-CABG, 244 sternotomy CABG), Nov 2015–Mar 2019	Multivessel CAD	MICS-CABG	Sternotomy CABG	Completeness of revascularization, 5-year MACCE, death, MI, stroke, repeat revascularization similar; cardiac outcomes equivalent	High graft patency; majority received ≥2 grafts.
Görtzen et al., 2024 [24]	Retrospective, PSM 1:2	210 patients after matching (73 Endo-CAB, 137 sternotomy), total cohort N=740	Total arterial multivessel CAD	Endoscopic-assisted MICS (Endo-CAB)	Sternotomy CABG	30-day mortality, re-exploration, ischemia/graft revision, CVA similar; AF lower in Endo-CAB; overall cardiac safety maintained	Higher textbook outcome, shorter hospital stay, and less blood loss.
Kakoush et al., 2025 [25]	Retrospective, single-center; matched cohort analysis	1915 patients (163 MIDCAB, 1752 conventional SITA CABG), 2000–2011	Yes – LAD-targeted multivessel CAD	MIDCAB	Conventional CABG using a single ITA	Early mortality and complications similar; long-term survival better in MIDCAB; cardiac events generally comparable in the matched cohort	Better long-term survival; similar early outcomes.
Bachinsky et al., 2012 [26]	Prospective comparative study	52 total (HCR n=25; OPCABG n=27)	Multivessel CAD (SYNTAX ≈ 34)	Same-sitting robotic-assisted HCR (MIDCAB + immediate PCI)	Off-pump CABG (OPCABG)	30-day MACE low; non-Q-wave MI lower in HCR; troponin lower in HCR; new AF similar; overall cardiac outcomes favorable for HCR	Much fewer transfusions; longer OR time; more OR extubations; shorter hospital & ICU stay; faster return to work.
Basman et al., 2020 [27]	Retrospective, propensity-matched	300 total (HCR=100; CABG=100; PCI=100) in triple-vessel disease (TVD)	Triple-vessel disease	HCR (LIMA-LAD + PCI for non-LAD)	CABG or multivessel PCI	Short-term 30-day mortality and MI similar; 8-year mortality and composite outcomes NS; overall cardiac outcomes comparable across groups	Shorter LOS; better residual SYNTAX; lower lesion-related complications.
Esteves et al., 2021 (MERGING trial) [28]	Pilot randomized controlled trial (2:1 allocation)	60 patients (HCR n=40; CABG n=20)	Complex triple-vessel CAD	Hybrid revascularization (LIMA-LAD + PCI for non-LAD vessels)	Conventional CABG	30-day and 1-year events slightly higher in HCR; 2-year composite MACE NS; cardiac outcomes generally comparable	Similar hospital stay; PCI had shortest recovery; higher stenosis in CABG; similar completeness of revascularization.
Ganyukov et al., 2020 (HREVS Trial) [29]	Randomized controlled trial (1:1:1)	155 total (CABG n=49; HCR n=49; PCI n=51) – multivessel CAD	Multivessel CAD suitable for all three strategies	HCR (MIDCAB LIMA-LAD + PCI to remaining vessels)	CABG and MV-PCI groups	Residual ischemia and 12-month MACCE similar among CABG, HCR, PCI; death, MI, stroke comparable; HCR non-inferior	HCR highly feasible; low conversion; moderate in-stent restenosis/occlusion.
Gąsior et al., 2014 (POL-MIDES/HYBRID Trial) [30]	Multicenter randomized controlled trial (1:1)	n=200 MVCAD patients (HCR=100, CABG=100)	MVCAD with LAD + ≥1 major vessel (>70% stenosis)	HCR (LIMA-LAD MIDCAB + PCI to non-LAD vessels)	Conventional CABG	In-hospital cardiac events low and similar; 12-month MACE similar; HCR feasible and safe	Shorter ventilation and hospital stay; higher angina-free survival.
Hage et al., 2019 (JAHA) [31]	Retrospective comparative study	n=363 (HCR=147; off-pump CABG=216)	Yes – MVCAD requiring LAD grafting + non-LAD vessel PCI	HCR (robotic MIDCAB LIMA-LAD + PCI to non-LAD)	Off-pump CABG	In-hospital MI, stroke, arrhythmia, death comparable; long-term survival higher in HCR; freedom from revascularization similar	Lower overall complications; lower transfusion; less drainage; more early discharge.
Harskamp et al., 2014 [32]	Propensity-matched cohort (1:4)	n=715 ≥65 yr (HCR=143; CABG=572)	Yes – MVCAD in elderly	HCR (MIDCAB LIMA-LAD + PCI)	CABG	30-day MACCE and 3-year mortality similar; cardiac outcomes comparable in elderly MVCAD patients	Higher bleeding in high-risk subgroup; renal outcomes similar.
Leacche et al., 2013 [33]	Retrospective cohort, stratified 2×2 by SYNTAX and EuroSCORE	n=381 CABG=301 HCR=80	Yes – MVCAD; complexity stratified as: SYNTAX ≤32 vs ≥33 • EuroSCORE <5 vs ≥5	HCR (LIMA-LAD + DES to non-LAD vessels)	CABG	Composite endpoint similar except high-risk subgroup (SYNTAX≥33 & EuroSCORE≥5) where HCR worse; other cardiac outcomes comparable	Excellent 5-year follow-up; no major outcome differences.
Tajstra et al., 2018 (POL-MIDES 5-year) [34]	Prospective randomized controlled trial	n=200 randomized (HCR=100, CABG=100) Analyzed at 5 years: n=191 (HCR=94, CABG=97)	Yes – MVCAD referred for surgery	HCR (MIDCAB LIMA-LAD + PCI)	CABG	5-year mortality, MI, repeat revascularization, stroke, MACCE all similar; cardiac outcomes comparable	Strong long-term follow-up; no major long-term differences.
Wu et al., 2017 [35]	Retrospective comparative cohort	HCR n=73 OPCAB n=383	Yes – MVCAD	2-staged HCR (MIDCAB LIMA-LAD + staged PCI)	OPCAB	Mid-term (~3 years) mortality, MI, neurologic events, repeat revascularization, MACCE all similar; cardiac outcomes comparable	Shorter operation and ventilation time; lower bleeding; fewer transfusions.

Quality Assessment

**Figure 2:**
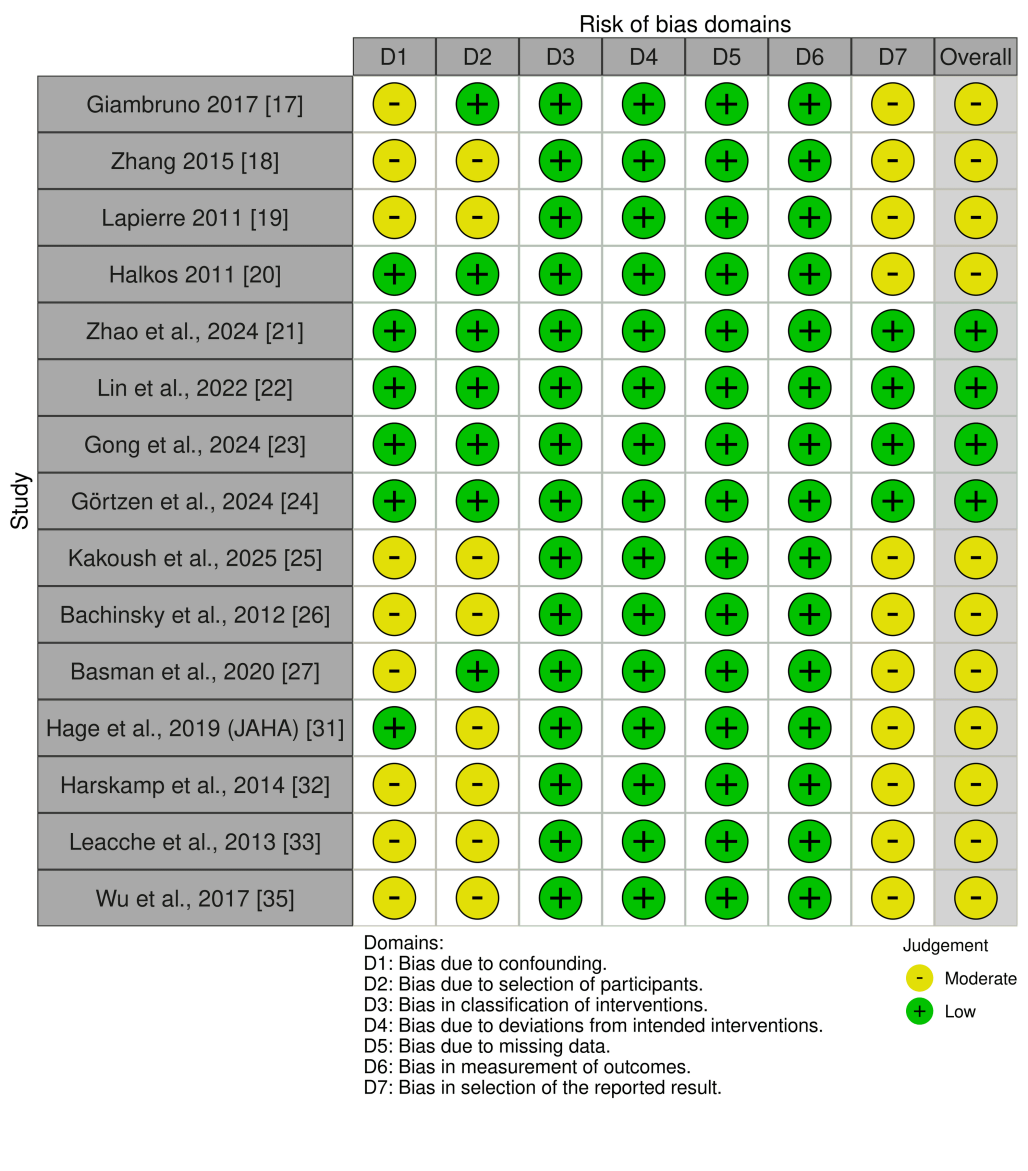
Quality assessment of non-randomized controlled trials by ROBINS-I Risk of bias in non-randomized studies assessed across seven domains: confounding, selection, classification of interventions, deviations from intended interventions, missing data, outcome measurement, and selective reporting. Each domain and overall bias is rated as Low, Moderate, Serious, Critical, or No Information (NI) [17-27, 31-33, 35]

Overall methodological quality varied across the included studies. Most non-randomized studies demonstrated moderate risk of bias, primarily due to confounding and participant selection limitations, although classification of interventions, deviations from intended procedures, missing data, and outcome measurement were generally well controlled. A few recent studies achieved low overall risk, reflecting stronger methodological rigor and appropriate adjustment for baseline differences (Figure 2) [21-24].

**Figure 3:**
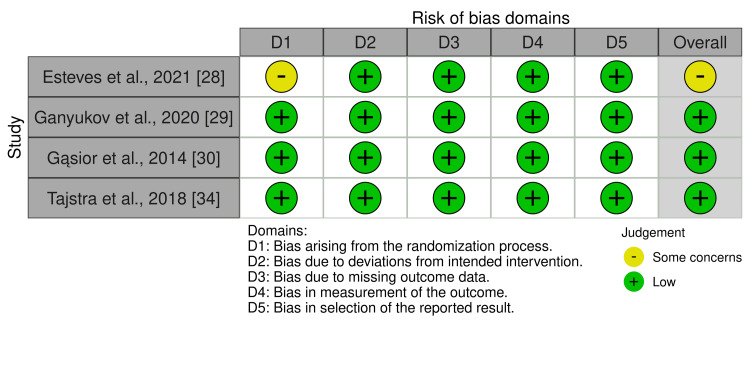
Quality assessment of RCTS by RoB 2.0. Risk of bias in randomized controlled trials assessed across five domains: bias arising from the randomization process, deviations from intended interventions, missing outcome data, measurement of the outcome, and selection of the reported result. Each domain and overall bias is rated as Low, Some Concerns, or High [28-30, 34].

Among RCTs, the risk of bias was low across most domains, with the HREVS trial, POL-MIDES, and POL-MIDES 5-year follow-up demonstrating consistently low risk [28-30,34]. The MERGING trial showed some concerns related to randomization but maintained low risk in other domains (Figure 3) [28].

Narrative synthesis

Mortality

Across the included studies, mortality rates were generally low and comparable between minimally invasive coronary revascularization and conventional CABG, but the reporting timepoints varied (in-hospital, 30-day, 1-year, 3-year, 5-year, and 8-year), and results were interpreted accordingly. Several cohorts reported early mortality (in-hospital or 30-day) of 0%-1% in both groups, including Zhang et al. (0.33% vs 0.85%; p=0.403), Zhao et al. (0% vs 0%), and Lin et al. (0.6% vs 0.6%) [18,21,22]. Where mortality was reported as a time-to-event outcome, Gong et al. showed no significant difference (10.6% vs 12.9%; HR 0.87; p=0.67) [23].

Robotic and hybrid studies showed similarly low early mortality in matched comparisons, including Halkos et al. (0.7% vs 0.9%; p=0.84) and Bachinsky et al. (0% vs 4%), although the latter was a small cohort (n=52) and should be interpreted cautiously [20,26]. Some studies suggested lower early mortality with minimally invasive techniques, such as Kakoush et al. (early 0.6% vs 3.6%; p=0.042; matched 0.7% vs 3.7%) [25]. In contrast, Giambruno et al. reported a small but statistically significant mortality difference favoring on-pump CABG (1.3% vs 0%; p=0.008), though the absolute event rate was very low and this cohort represented LAD + one-vessel multivessel disease rather than uniformly “high-complexity” anatomy [17].

Randomized trials accounted for longer follow-up comparisons. POL-MIDES reported no mortality difference at 12 months, and its linked 5-year follow-up (Tajstra et al.) also demonstrated no significant difference (6.4% vs 9.2%; p=0.69) [30,34]. Similarly, the HREVS trial reported comparable mortality at 12 months (2% vs 5.8%; p=0.78) across CABG/HCR/PCI arms [29]. Across studies that stratified baseline risk (e.g., Leacche et al. by SYNTAX and EuroSCORE), directional mortality differences were most apparent in higher-risk subgroups, suggesting that baseline complexity and surgical risk may partly explain outcome variation rather than approach alone [33].

Analytically, studies where HCR was compared with multivessel PCI (e.g., Basman et al., HREVS trial) were interpreted separately from the primary minimally invasive vs conventional CABG comparison to avoid dilution of the core surgical comparator.

Myocardial Infarction

Rates of MI were consistently similar between groups, although MI definitions and detection thresholds were variably reported across studies. In Zhang et al., MI occurred in 0.67% of minimally invasive patients versus 0.56% with OPCAB (p=0.124) [18]. Comparable neutral findings were reported in Lin et al. (1.7% vs 1.2%; p=0.657), Gong et al. (5.6% vs 4.2%; p=0.73), and Giambruno et al. (1.1% vs 1.4%; p=0.53) [17,22,23]. Halkos et al. similarly found low rates (0.7% vs 0.5%; p=0.8) [20].

A notable outlier was Bachinsky et al., reporting markedly high non-Q-wave MI rates (60% vs 92%; p=0.009) despite 0% Q-wave MI in both arms [26]. Given the magnitude of these event rates and the simultaneous reporting of lower postoperative troponin in HCR, these findings likely reflect intensive perioperative biomarker surveillance and a low reporting threshold for enzyme-defined infarction, rather than clinically adjudicated infarction comparable to other cohorts. Long-term MI rates remained similar in POL-MIDES 5-year follow-up (4.3% vs 7.2%; p=0.30) and were comparable across strategies in HREVS (8% vs 5.8%; p=0.66) [29,34]. Where reported, MI outcomes predominantly reflected perioperative or early events, and late/spontaneous MI was not consistently distinguishable across studies.

Stroke

Stroke was infrequently reported and was predominantly measured as a perioperative or early neurologic complication, limiting inference regarding longer-term neurologic outcomes. Halkos et al. reported identical stroke rates (0.7% vs 0.7%; p=1.0) [20]. Lin et al. and Gong et al. similarly demonstrated low rates (0.6% vs 0.6% and 6.7% vs 6.6%, respectively; p=0.83 in Gong) [22,23]. Giambruno et al. reported 2.4% versus 2.1% (p=0.22), and Zhao et al. reported 5.8% versus 3.9% (p=0.663) [17,21]. Endoscopic CAB was numerically associated with fewer cerebrovascular events in Görtzen et al. (0% vs 1.5%), but this was not statistically significant [24].

Transient ischemic attacks (TIAs) were not consistently reported as part of stroke outcomes, and stroke may be underreported in studies where it was not a primary endpoint or where ascertainment relied on administrative/clinical records rather than formal adjudication. Overall, available data suggest comparable early neurologic safety between approaches.

Atrial Fibrillation

Postoperative AF varied across studies but generally showed mixed trends. Several analyses demonstrated similar AF rates between groups, including Giambruno et al. (15% vs 12%; p=0.12), Lapierre et al. (23% vs 28%; p=0.3), and Lin et al. (20.4% vs 19.8%; p=0.904) [17,19,22]. Endoscopic CAB demonstrated significantly lower AF in Görtzen et al. (5.5% vs 17.5%; p=0.015) [24]. Bachinsky et al. observed 16% versus 30% (p=0.329) [26].

Across studies, AF was typically reported as postoperative/new-onset AF during the index admission; recurrence of pre-existing AF and post-discharge AF surveillance were not consistently captured. The observed reduction in AF in highly minimally invasive approaches such as Endo-CAB is biologically plausible, potentially reflecting differences in surgical access, extent of pericardial manipulation, reduced inflammatory response, and avoidance of cardiopulmonary bypass in selected cohorts, although these mechanisms could not be tested directly within included designs.

Repeat Revascularization

Repeat revascularization was infrequent but appeared higher in some HCR cohorts, reflecting the PCI component and potential for restenosis or progression in stented segments. Halkos et al. reported significantly higher repeat revascularization in HCR versus OPCAB (12.2% vs 3.7%; p<0.001) [20]. In contrast, Giambruno et al. found similar long-term revascularization (7% vs 9%; p=0.27), and Lin et al. reported low and comparable rates (1.2% vs 2.3%; p=0.423) [17,22]. Gong et al. and Wu et al. also reported near-identical rates (1.9% vs 1.8% and 1.4% vs 1.7%, respectively) [23,35].

Longer-term revascularization was substantial in the POL-MIDES 5-year dataset (37.2% vs 45.4%; p=0.38) [34]. However, repeat revascularization definitions varied across studies (target-vessel revascularization vs any repeat coronary intervention), and early technical failure (e.g., early graft occlusion reported as 1.2% in Zhang et al.) could not always be separated from later disease progression-driven reintervention [18]. Follow-up completeness and surveillance intensity may also differ between hybrid and surgical-only cohorts, particularly where routine angiographic follow-up was performed.

Composite Major Adverse Cardiac and Cerebrovascular Events (MACE/MACCE)

Composite outcomes were broadly similar across minimally invasive and conventional approaches, but direct comparison is limited by variability in composite endpoint definitions and follow-up duration. Zhao et al. reported comparable MACCE (7.7% vs 5.9%; p=0.715) [21], while the HREVS trial reported similar 12-month MACCE (13.4% vs 12%; p=0.83) across CABG/HCR/PCI [21,29]. Halkos et al. reported identical in-hospital MACCE (2% vs 2%) [20]. Gong et al. demonstrated similar 5-year MACCE (19.9% vs 22.1%; HR 0.80; p=0.39), and POL-MIDES reported non-significant differences at 12 months and at 5 years (45.2% vs 53.4%; p=0.39) [23,30,34].

Subgroup findings should be interpreted cautiously. Leacche et al. reported worse composite outcomes for HCR in the high-risk subgroup defined by SYNTAX ≥33 and EuroSCORE ≥5, indicating that baseline complexity and operative risk may modify comparative effectiveness [33]. Giambruno et al. reported a clinically meaningful difference in angina-free survival favoring conventional CABG (70% vs 91%; p<0.001) despite otherwise similar cardiac events (Table 2) [17]. Overall equivalence conclusions were therefore framed with explicit recognition of risk-stratified subgroup signals and variable composite endpoint composition.

**Table 2:** Statistical results of cardiac outcomes. MIDCABG: minimally invasive direct CABG; MICS-CABG: minimally invasive CABG; HCR: hybrid coronary revascularization; PCI: percutaneous coronary intervention; LITA: left internal thoracic artery; LAD: left anterior descending; OPCAB: off-pump CABG; SITA: single internal thoracic artery; MACCE/MACE: major adverse cardiac/cerebrovascular events; NR: not reported; HR: hazard ratio; NS: not significant; TVR: target vessel revascularization; CVA: Stroke Outcomes: Mortality, MI, Stroke, AF – Atrial Fibrillation, Repeat Revascularization, Composite (MACCE/MACE). Percentages shown as Intervention vs Control; p-values reported when available.

Author/Year	Intervention vs Control	Mortality	MI	Stroke	Atrial Fibrillation	Repeat Revascularization	Composite Outcomes (MACCE/MACE)
Giambruno et al. 2017 [17]	Robotic MIDCAB (LITA-LAD) + PCI vs On-pump CABG	1.3% vs 0% (p=0.008)	1.1% vs 1.4% (p=0.53)	2.4% vs 2.1% (p=0.22)	15% vs 12% (p=0.12)	7% vs 9% long-term (p=0.27)	Long-term angina-free survival: 70% vs 91% (p<0.001)
Zhang et al. 2015 [18]	MIDCAB (hybrid/single LAD/MVD) vs OPCAB sternotomy	0.33% vs 0.85% (p=0.403)	0.67% vs 0.56% (p=0.124)	NR	NR	Graft occlusion early: 1.2%	NR
Lapierre et al. 2011 [19]	MICS-CABG (thoracotomy) vs OPCAB sternotomy	0% vs 0%	NR	NR	23% vs 28% (p=0.3)	NR	NR
Halkos et al. 2011 [20]	HCR (LIMA-LAD + PCI) vs OPCAB sternotomy	0.7% vs 0.9% (p=0.84)	0.7% vs 0.5% (p=0.8)	0.7% vs 0.7% (p=1.0)	NR	12.2% vs 3.7% (p<0.001)	In-hospital MACCE 2% vs 2%
Guangxin et al. 2024 [21]	MICS-CABG vs Conventional CABG (sternotomy)	0% vs 0%	1.9% vs 2.0% (p=0.989)	5.8% vs 3.9% (p=0.663)	NR	0% vs 0%	MACCE 7.7% vs 5.9% (p=0.715)
Liang et al. 2022 [22]	MICS-CABG vs OPCAB sternotomy	0.6% vs 0.6%	1.7% vs 1.2% (p=0.657)	0.6% vs 0.6%	20.4% vs 19.8% (p=0.904)	1.2% vs 2.3% (p=0.423)	NR
Gong et al. 2024 [23]	MICS-CABG vs Sternotomy CABG	10.6% vs 12.9% (HR 0.87; p=0.67)	5.6% vs 4.2% (p=0.73)	6.7% vs 6.6% (p=0.83)	NR	1.9% vs 1.8%	5-year MACCE 19.9% vs 22.1% (HR 0.80; p=0.39)
Görtzen et al. 2024 [24]	Endo-CAB (Endoscopic) vs Sternotomy CABG	1.4% vs 0.7% (p=0.655)	Postop ischemia 0% vs 0.7%	CVA 0% vs 1.5%	5.5% vs 17.5% (p=0.015)	NR	NR
Kakoush et al. 2025 [25]	MIDCAB vs Conventional SITA-CABG	Early: 0.6% vs 3.6% (p=0.042) Matched: 0.7% vs 3.7%	1.5% vs 1.2% (p=0.764)	1.8% vs 1.8%	NR	NR	Long-term survival better with MIDCAB (HR 0.559; p=0.004)
Bachinsky et al. 2012 [26]	Robotic HCR (MIDCAB + PCI) vs OPCAB	0% vs 4%	Q-wave: 0 vs 0; Non-Q-wave: 60% vs 92% (p=0.009)	0 vs 0	16% vs 30% (p=0.329)	0 vs 0	30-day MACE 0% vs 4%
Basman et al. 2020 [27]	HCR vs CABG vs PCI	8-year: 5% vs 4% vs 9% (NS)	8-year composite death/MI: HCR 21% vs CABG 15%	NR	NR	Revasc: HCR 21% vs CABG 15% vs PCI 25%	8-year composite 21% vs 15% (p=0.36)
Esteves et al. 2021 [28] (MERGING)	HCR vs CABG	30-d: 5% vs 0%; 1-year: 5% vs 0%	30-d: 10% vs 0%; 1-year: 12.5% vs 5.9%	0% vs 0%	NR	30-d: 7.6% vs 0%; 1-year: 10.6% vs 5.9%	2-year MACE 19.3% vs 5.9%
Ganyukov et al. 2020 [29] (HREVS)	HCR vs CABG vs PCI	2% vs 5.8% (p=0.78)	8% vs 5.8% (p=0.66)	0% vs 3.8% (p=0.21)	NR	TVR clinical: 2% vs 1.9%	12-mo MACCE 13.4% vs 12% (p=0.83)
Gąsior et al. 2014 [30](POL-MIDES)	HCR vs CABG	0% vs 0%	5.1% vs 3.9% (p=0.69)	0% vs 0%	NR	2% vs 0%	12-mo MACE similar (NS)
Hage et al. 2019 [31]	HCR vs OPCAB	0% vs 1% (p=0.15)	1.4% vs 0.5% (p=0.36)	2.1% vs 1% (p=0.88)	12% vs 19% (p=0.13)	3.4% vs 0% (p=0.03)	Long-term survival 96% vs 85% (p=0.054)
Harskamp et al. 2014 [32]	HCR vs CABG	30-d: 5.6% vs 3.8%; 3-yr: 13.2% vs 16.6%	NS	NS	NR	NR	30-d MACCE: 5.6% vs 3.8%
Leacche et al. 2013 [33]	HCR vs CABG	Higher in high-risk HCR (33% vs 0%)	MI similar	Stroke similar	NR	NR	Composite worse in SYNTAX≥33 + EuroSCORE≥5 subgroup
Tajstra et al. 2018 [34]	HCR vs CABG	5-yr: 6.4% vs 9.2% (p=0.69)	4.3% vs 7.2% (p=0.30)	2.1% vs 4.1% (p=0.35)	NR	37.2% vs 45.4% (p=0.38)	5-yr MACCE 45.2% vs 53.4% (p=0.39)
Wu et al. 2017 [35]	2-stage HCR vs OPCAB	1.4% vs 0.3% (p=0.309)	4.1% vs 2.2% (p=0.598)	0% vs 0.3%	NR	1.4% vs 1.7%	MACCE 6.8% vs 4.4% (p=0.269)

Operative Metrics and Hospital Course

Operative metrics varied by technique. MIDCAB studies often showed shorter operative durations compared with sternotomy OPCAB in Zhang et al. (152 vs 263 min) and Wu et al. (153 vs 263 min) [18,35]. Conversely, robotic/hybrid procedures were associated with longer durations (e.g., Bachinsky et al. 386 vs 261 min), likely reflecting procedure complexity and learning-curve effects [26]. Operative time definitions were not standardized across studies (skin-to-skin vs total OR time), and therefore were interpreted descriptively as reported.

Blood loss was generally lower in minimally invasive cohorts, though measurement varied (intraoperative loss vs cumulative perioperative drainage). Zhao et al. reported 600 vs 700 mL, and Görtzen et al. reported 360 vs 490 mL [21,24]. Wu et al. reported lower bleeding in staged HCR versus OPCAB (559 vs 1035 mL) and markedly fewer transfusions (16% vs 52%) [35]. Hospital course consistently favored minimally invasive approaches, with shorter LOS reported in multiple cohorts (e.g., Giambruno 4.5 vs 6.7 days; Lapierre 5.4 vs 7.2 days; Görtzen 4 vs 6 days; Hage 4.5 vs 8.1 days) [17,19,24,31]. ICU and ventilation times were also shorter in several studies (Zhang: ventilation 9.27 vs 24.9 h; ICU 24.3 vs 59.1 h; Wu: ventilation 9.4 vs 19 h) [18,35]. These hospital-course outcomes may partly reflect institutional protocols and pathway differences in addition to physiologic recovery, and conversion events were reported descriptively without consistent stratified analysis.

Functional Recovery and Angina

Functional recovery and angina outcomes were generally favorable in minimally invasive cohorts, but instruments and timing of assessment were heterogeneous. Lin et al. reported improved activities of daily living using the Barthel Index (51.8 ± 21.2 vs 41.7 ± 21.6; p<0.001) [22]. Hage et al. reported higher angina-free survival in HCR compared with OPCAB (90% vs 73%), whereas Giambruno et al. reported lower angina-free survival in HCR compared with on-pump CABG (70% vs 91%; p<0.001) [17,31]. Lapierre et al. and Bachinsky et al. described faster return to work and daily activity (p<0.01), though these outcomes were not uniformly adjudicated and were commonly derived from clinical documentation or patient-reported recovery milestones (Table 3) [19,26].

**Table 3:** Statistical results of survival outcomes. MIDCABG: Minimally Invasive Direct CABG; MICS-CABG: Minimally Invasive CABG; HCR: hybrid coronary revascularization; PCI: percutaneous coronary intervention; OPCAB: off-pump CABG; SITA: single internal thoracic artery; OR: operating room; LOS: length of stay; ICU: intensive care unit; AF: atrial fibrillation; RBC: red blood cell transfusion. Metrics: Operative time, hospital course (LOS, ICU stay, ventilation), complications, graft patency, functional recovery, and other procedure-specific outcomes.

Author/Year	Intervention vs Control	Operative Metrics	Hospital Course (LOS / ICU / Ventilation)	Complications / Other Findings
Giambruno et al. 2017 [17]	Robotic MIDCAB + PCI vs On-pump CABG	—	LOS: 4.5 vs 6.7 days (p<0.001)	Less ventilation >24h; no difference in dialysis or transfusion
Zhang et al. 2015 [18]	MIDCAB (mixed) vs OPCAB	OR time: 152 vs 263 min	Ventilation: 9.27 vs 24.9 h; ICU: 24.3 vs 59.1 h	RBC transfusion: 0.79 vs 3.26 U
Lapierre et al. 2011 [19]	MICS-CABG vs OPCAB	—	LOS: 5.4 vs 7.2 days	Wound infection: 0% vs 4%; pleural effusion: 15% vs 4%; faster return to activity
Halkos et al. 2011 [20]	HCR vs OPCAB	—	LOS similar	Transfusion: 35% vs 56%
Zhao et al. 2024 [21]	MICS-CABG vs Conventional	OR duration slightly longer	—	Blood loss: 600 vs 700 mL; wound debridement: 5.8% vs 19%
Liang et al. 2022 [22]	MICS-CABG vs OPCAB	OR duration longer in MICS	LOS: 6.2 vs 6.9 days	ADL functional recovery better (Barthel score improved)
Gong et al. 2024 [23]	MICS-CABG vs Sternotomy CABG	—	—	Bypassed vessels: 2–4; graft patency similar
Görtzen et al. 2024 [24]	Endo-CAB vs Sternotomy	—	LOS: 4 vs 6 days; Blood loss: 360 vs 490 mL	Textbook outcome achieved: 78% vs 59%; AF lower: 5.5% vs 17.5%
Kakoush et al. 2025 [25]	MIDCAB vs Conventional SITA CABG	—	—	Long-term survival better in MIDCAB; early outcomes similar
Bachinsky et al. 2012 [26]	Robotic HCR vs OPCAB	OR time: 386 vs 261 min	Extubation in OR: 68% vs 22%; LOS: 5.1 vs 8.2 days; ICU: 28 vs 58 h	Transfusion: 12% vs 67%
Basman et al. 2020 [27]	HCR vs CABG vs PCI	—	LOS: 5.7 vs 7.5 vs 2.0 days	Residual SYNTAX lower in HCR (4.5 vs PCI 7.1)
Esteves et al. 2021 [28](MERGING RCT)	HCR vs CABG	—	—	Procedure-specific lesion failure: CABG 9.5% vs HCR 2.6%
Ganyukov et al. 2020 [29] (HREVS RCT)	HCR vs CABG vs PCI	—	LOS: PCI 4.5 days, CABG 13.8 days	Sick leave: 23 vs 16 vs 8 weeks; angiographic stenosis highest in CABG
Gąsior et al. 2014 [30] (POL-MIDES)	HCR vs CABG	—	—	In-stent restenosis 7.5%; conversion from HCR 6%
Hage et al. 2019 [31]	HCR vs OPCAB	—	LOS: 4.5 vs 8.1 days; ventilation >24h lower	More freedom from angina in HCR (90% vs 73%)
Harskamp et al. 2014 [32]	HCR vs CABG (elderly)	—	LOS <5 days: 46% vs 27%	Complications: 9% vs 18%; transfusion: 28% vs 53%
Leacche et al. 2013 [33]	HCR vs CABG	—	—	High-risk group bleeding: 44% vs 11%
Tajstra et al. 2018 [34](5-yr POL-MIDES)	HCR vs CABG	—	—	No major differences at 5 years
Wu et al. 2017 [35]	Staged HCR vs OPCAB	OR time: 153 vs 263 min	Ventilation: 9.4 vs 19 h; Bleeding: 559 vs 1035 mL	Transfusion: 16% vs 52%

Differences in completeness of revascularization and residual ischemia (including residual SYNTAX metrics where reported, e.g., Basman et al.) may contribute to variability in angina-related outcomes, particularly in hybrid strategies where non-LAD lesions are treated with PCI [27]. Overall, the data suggest minimally invasive approaches can accelerate recovery and improve patient-centered outcomes, while symptom outcomes may be sensitive to revascularization completeness and follow-up ascertainment.

Discussion

The evidence synthesized in this review indicates that minimally invasive coronary revascularization techniques, when considered by a specific approach, can achieve cardiac outcomes comparable to conventional sternotomy-based CABG in selected patients with multivessel or otherwise complex CAD. Importantly, equivalence should not be overgeneralized across all minimally invasive strategies, as the included evidence spans distinct procedures (MIDCAB, multivessel MICS-CABG, endoscopic/endo-assisted CAB, and HCR) with different technical goals, completeness-of-revascularization profiles, and follow-up patterns. Overall, the dominant pattern across cohorts was low early event rates and broadly similar safety outcomes, but the strength of inference varied by technique, patient selection, and study design.

MIDCAB and minimally invasive multivessel CABG (MICS-CABG / thoracotomy-based multivessel bypass) demonstrated consistently favorable perioperative profiles and generally similar early cardiac outcomes compared with sternotomy CABG or sternotomy OPCAB. In mixed and matched cohorts, early mortality and major complications were comparable (e.g., Zhang et al. and Lapierre et al.), while recovery advantages-shorter ventilation and ICU duration, fewer transfusions, and shorter length of stay-were frequently reported [18], [19]. In the large matched MIDCAB cohort (Kakoush et al.), early mortality was lower, and long-term survival was reported as improved, supporting the possibility that selected LAD-targeted multivessel candidates may benefit from a less invasive strategy without compromising durability [25]. However, MIDCAB evidence also includes mixed anatomical populations (e.g., incomplete multivessel cohorts in Zhang et al.), underscoring that careful attention to CAD complexity is necessary when interpreting generalizability [18].

Endoscopic/endo-assisted CAB (Endo-CAB) was represented by fewer studies but showed encouraging signals. In Görtzen et al., early mortality and cerebrovascular events were similar to sternotomy CABG, while postoperative AF was significantly lower (5.5% vs 17.5%), and “textbook outcome” rates were higher [24]. These findings suggest that highly minimally invasive approaches may reduce arrhythmia-related morbidity and accelerate recovery, although broader validation is needed given limited study volume and the likelihood that these procedures are concentrated in specialized centers [24].

HCR showed the most variable pattern because it combines surgical LIMA-LAD grafting with PCI for non-LAD disease. Across multiple studies, early mortality, MI, and stroke were generally comparable to CABG, including in matched cohorts and randomized trials [20,29,30,34]. However, repeat revascularization signals were more mixed in HCR than in surgical-only minimally invasive strategies, with some cohorts showing higher target-vessel revascularization (e.g., Halkos et al. 12.2% vs 3.7%; p<0.001) [20], while others showed similar rates (e.g., Giambruno et al., Gong et al., Wu et al.) [17], [23], [35]. Clinically, these differences likely reflect PCI durability (in-stent restenosis, progression in non-LAD segments), staging strategy (same-sitting vs staged HCR), and surveillance intensity, rather than failure of the minimally invasive LIMA-LAD component itself. This interpretation is supported by the observation that many studies report comparable survival despite occasional reintervention differences, implying that HCR trade-offs may center on reintervention burden and patient experience rather than mortality.

Across the literature, short-term outcomes (in-hospital/30-day) consistently demonstrated low event rates and broad safety equivalence between minimally invasive strategies and conventional CABG in appropriately selected patients [18-22,24,26]. Mid-term outcomes (≈12 months to 3 years)-available in trials such as POL-MIDES (12-month), HREVS (12-month), and observational cohorts, also suggested similar composite endpoints and survival [29,30,32,35]. Long-term outcomes (5-8 years) were more variably reported but remained broadly consistent with equivalence in randomized follow-up (POL-MIDES 5-year) and selected cohorts, with occasional directional differences by subgroup and technique [34]. Therefore, conclusions of “equivalence” are most robust for early safety endpoints, reasonably supported for mid-term outcomes, and more conditional for long-term outcomes due to variable follow-up completeness and endpoint definitions across studies.

A substantial proportion of included evidence derives from retrospective propensity-matched or weighted cohorts. Although propensity approaches reduce measured baseline imbalance, they cannot fully eliminate selection bias, referral bias, and unmeasured confounding (e.g., frailty, coronary diffuseness, conduit quality, surgeon preference). Consequently, part of the observed equivalence-especially for low-frequency endpoints such as early mortality-may reflect careful selection of patients deemed suitable for minimally invasive or hybrid approaches, rather than pure procedural equivalence. This is clinically important because minimally invasive strategies are frequently offered in centers with structured selection pathways, potentially enriching for patients with favorable anatomy or risk profiles.

Risk stratification emerged as a key modifier of outcomes. Anatomical complexity and clinical risk (SYNTAX score and EuroSCORE) appear particularly relevant for HCR suitability, as illustrated by Leacche et al., where outcomes were worse in the highest-risk subgroup (SYNTAX ≥33 and EuroSCORE ≥5) [33]. This supports a selective role for HCR, potentially favoring lower-to-intermediate complexity anatomy, while conventional CABG may remain preferable in very high anatomical/clinical risk combinations. Patient factors also matter: several cohorts included elderly or diabetic populations (e.g., Harskamp et al., Zhao et al.), suggesting feasibility in higher-risk groups, but evidence remains heterogeneous and technique-dependent [21,32]. Future studies and clinical pathways should incorporate structured selection using anatomical complexity (e.g., SYNTAX category), surgical risk scores, and comorbidity profiles (diabetes, age) when choosing between minimally invasive CABG variants, HCR, and conventional CABG.

Beyond “hard” cardiac endpoints, minimally invasive strategies consistently improved perioperative recovery metrics, including shorter ventilation time, reduced ICU utilization, shorter length of stay, and lower transfusion requirements in multiple cohorts [17-19,24,35]. These advantages are clinically meaningful because they translate into reduced early morbidity, faster mobilization, and potentially earlier return to normal activity. This is supported by functional recovery findings in studies using patient-centered assessments, such as improved Barthel Index activities-of-daily-living scores (Lin et al.) and improved angina-related outcomes in some cohorts (Hage et al.) [22,31]. However, quality-of-life and functional recovery endpoints were not standardized across studies (instrument choice, timing, and ascertainment varied), limiting cross-study comparability and precluding strong conclusions beyond a general signal of faster recovery.

A major interpretive limitation is the absence of uniformly standardized “cardiac functional” endpoints across studies. Most included studies reported clinical event outcomes (mortality, MI, stroke, AF, revascularization, MACCE/MACE) rather than consistent physiologic surrogates (e.g., LVEF, HF functional class, ventricular remodeling). As a result, interpretation is most confident for safety and morbidity endpoints, whereas inference regarding true cardiac functional recovery (in a physiologic sense) remains limited. This reinforces the need for future comparative trials to incorporate standardized patient-reported outcomes and functional metrics, in addition to clinical events and graft/PCI durability measures.

Another important consideration is that many minimally invasive and hybrid procedures are performed in specialized, high-volume centers. Learning-curve effects and institutional volume likely influence operative time, conversion rates, completeness of revascularization, and postoperative outcomes, particularly for robotic and endoscopic techniques. This may explain why some cohorts show pronounced recovery advantages without safety trade-offs. Accordingly, external validity may be limited when translating results to lower-volume settings or early program adoption, and comparative effectiveness may differ outside experienced centers.

These findings are consistent with contemporary guideline-based frameworks that favor CABG in high anatomical complexity disease (e.g., left-main and/or high SYNTAX), while recognizing roles for PCI and hybrid strategies in selected anatomies and patient contexts. The present review suggests that, within appropriately selected multivessel/complex CAD populations, minimally invasive surgical approaches-particularly MIDCAB/MICS-CABG and carefully selected HCR-may offer recovery and patient-centered advantages without clear early safety penalties, while acknowledging that repeat revascularization risk may be higher in some HCR cohorts because of PCI durability and staging considerations. Thus, the clinical implication is not universal substitution of conventional CABG, but refined patient selection and shared decision-making informed by anatomy, risk profile, institutional expertise, and patient preferences regarding recovery versus potential reintervention.

This review has several limitations. First, the evidence base is dominated by retrospective and propensity-matched observational studies, with relatively few RCTs, which limits causal inference and introduces potential residual confounding. Considerable clinical and methodological heterogeneity was present across studies, including variations in minimally invasive techniques, surgeon expertise, patient selection criteria, and definitions of outcomes such as MACCE and repeat revascularization. Many studies included mixed populations rather than exclusively complex or multivessel CAD, reducing the precision of comparisons for high-complexity cohorts. Several minimally invasive cohorts were drawn from high-volume centers with specialized expertise, limiting generalizability. Follow-up duration varied widely, and long-term graft patency and survival data were inconsistently reported. Finally, the inability to perform a meta-analysis restricts quantitative synthesis. Future work should prioritize multicenter randomized trials and pragmatic registries with standardized endpoint definitions, explicit complexity stratification (SYNTAX/EuroSCORE), structured reporting of completeness of revascularization, and standardized quality-of-life instruments to capture recovery and patient-centered benefit.

## Conclusions

Minimally invasive coronary revascularization, particularly MIDCAB, multivessel MICS-CABG, and HCR in selected cohorts, demonstrates cardiac outcomes comparable to conventional sternotomy CABG in appropriately selected patients with multivessel or complex CAD, while consistently offering perioperative advantages such as shorter ICU and hospital stay, reduced transfusion requirements, and faster recovery. Appropriateness of selection most commonly reflects patients with LAD disease plus ≥1 additional vessel amenable to complete minimally invasive revascularization and without combined very-high anatomical and clinical risk (e.g., SYNTAX ≥33 with EuroSCORE ≥5). Across short- and mid-term follow-up, mortality, MI, stroke, and MACCE/MACE rates remain broadly similar, although some HCR cohorts show higher repeat revascularization-likely driven by PCI durability rather than failure of the LIMA-LAD graft-highlighting the need for shared decision-making. Evidence strength varies by technique, with robotic and endoscopic approaches supported by fewer studies and more influenced by learning-curve and center-volume effects; thus, conclusions are most applicable to high-volume, experienced centers. Future work should focus on multicenter randomized trials in high-complexity strata, standardized definitions of outcomes and revascularization completeness, and uniform patient-centered and functional outcome reporting.
